# Porous Clay Heterostructure with Alginate Encapsulation for Toluene Removal

**DOI:** 10.3390/nano11020388

**Published:** 2021-02-03

**Authors:** Yeongkyun Son, Tae-Hyun Kim, Daekeun Kim, Yuhoon Hwang

**Affiliations:** Department of Environmental Engineering, Seoul National University of Science and Technology, Seoul 01811, Korea; clayton7167@seoultech.ac.kr (Y.S.); th.kim@seoultech.ac.kr (T.-H.K.); kimd@seoultech.ac.kr (D.K.)

**Keywords:** bentonite, porous clay heterostructure, volatile organic carbon, toluene, adsorption–desorption

## Abstract

A volatile organic compound adsorbent based on a porous clay heterostructure (PCH) with alginate biopolymer was successfully prepared. From N_2_ adsorption–desorption analysis, the specific surface area, pore volume, and pore size of bentonite were dramatically increased after introducing the porous structure. Following complexation with alginate (Alg-PCH), the pore volume and pore size were not significantly affected by pore structure. The thermal stability of Alg-PCH shows enhanced thermal stability compared to alginate and alginate beads. The morphology layered structure of Alg-PCH was carried out by transmission electron microscopy (TEM), suggesting the disorder and re-order of the *c*-axis layer stacking by porous structure and complexation with alginate, respectively, which was well-matched with X-ray diffraction results. To optimize the preparation of Alg-PCH, various reaction conditions (alginate, CaCl_2_ concentration, bead size, and weight ratio between alginate and PCH) were utilized. According to the toluene adsorption–desorption experiments, the preparation conditions for Alg-PCH were selected as a 2 mm extrusion tip, 0.5% of alginate, and 2% of CaCl_2_ solution with a 1:50 alginate:PCH weight ratio. Additionally, it shows 61.63 mg/g adsorption capacity with around 49% desorption efficacy under atmospheric temperature and pressure.

## 1. Introduction

In recent decades, air pollution due to the development of many anthropogenic sources in vehicle exhaustion, oil gas evaporation, industry emission, and waste material disposal has been an emerging global issue. Volatile organic compounds (VOCs) are air pollutants defined as a liquids or gases that evaporate easily into the atmosphere due to high vapor pressure (0.1 mm Hg) [1,2,3]. In particular, VOCs such as benzene, toluene, and xylene have been regulated due to them causing cancer or odor [4,5,6]. In the Republic of Korea, VOCs are among the main air pollutants, and around 55% of secondary atmospheric particulate matter is derived from organic compounds, such as toluene [7,8,9]. The VOCs are transformed in the air into secondary atmospheric particulate matter via photochemical reaction, leading to environment pollution. Additionally, when coexisting with nitrogen oxide (NO_x_), VOCs can produce photochemical products such as ozone (O_3_) and peroxyacetyl nitrate (PAN), causing smog and global warming [10]. In addition to the environmental risk, VOCs can also significantly affect human health, such as eye irritation, confusion, nausea, asthma, and carcinogenic and mutagenic problems [11,12,13]. 

Most VOCs are attributed to organic solvents widely used in painting, laundry, construction, pavements, etc. [7,9,14]. Most of these places are nearby residential areas; therefore, the mitigation technology for VOC removal is very important. As an example, laundry facilities produce VOCs during dry cleaning processes, and these facilities can be easily found in cities. Due to the fast discharge of VOCs into the gas phase, removal of VOCs before discharge into the air is the key to reducing VOCs in the air system. Many studies on VOC removal have been conducted, such as on adsorption [15,16], biofilter [17], cooling condensation [18], and catalytic incineration [19]. Among those techniques, adsorption could be promising due to its easy operation, low cost, use at low concentrations, and regeneration of adsorbents through the adsorption and desorption process [20,21]. Furthermore, reusable adsorbents make it possible to save on economic costs and could be sufficiently applied even in small sites, such as laundry facilities [22]. 

Activated carbon has been most widely studied for VOC adsorption due to its high specific surface area (SSA) and hydrophobicity [15,23,24]. However, application of activated carbon is limited by poor thermal stability and difficulty of regeneration due to its thermal and chemical instability [25,26,27]. Furthermore, the desorption process of activated carbon is strongly dependent on the temperature and pressure conditions [28,29]. Another adsorbent, hydrophobic zeolite, has also been frequently studied for VOC adsorption, but zeolite is expensive and sensitive to humidity [30]. Clay materials, which are easily found in nature, also have a unique layered structure, cation exchange capacity, and high thermal stability with an inexpensive price [31,32,33]. Due to the characteristic features of clay materials, they have been studied as adsorbents for heavy metal, organic compounds, and humic acids [34,35,36,37]. However, clay materials show lower adsorption capacity on VOCs than activated carbon [27]. In order to use clay materials as adsorbents, porous clay heterostructures (PCHs) have recently emerged, which increase hydrophobicity by introducing surfactant and maximize internal pore volume by using an organosilicon in the interlayer space [31,38]. Furthermore, PCH has large SSA and pore volume as a result of being composed of silicon pillars, and introduced Si enhanced thermal stability compared to other adsorbents, which makes it possible to utilize it for high-temperature emission gases [39]. 

In order to evaluate the adsorption performance study for practical application, granule form could be easier to handle than powdered samples. The VOC adsorption with powdered samples might lead to clogging as the pressure drops and following mechanical damage and explosions [40]. Many granulation studies have been conducted, and the pellet molding method by strong compressive force is mostly used due to its simple preparation requirements. However, the price of machines for pellet molding is high, and pelletizing pressure might affect the internal structure and pore properties [41,42]. To overcome the disadvantages of pelletizing, the preparation of polymer-based granular substances has been conducted [43,44,45,46]. Among the various polymers, alginate has attracted attention due to being an environmentally friendly polymer with a compatible cheap price, as it is extracted from brown algae and is easy to make beads from by simply dropping it on a crosslinking agent, such as multi-valent cations [47]. The alginate encapsulation usually forms sphere-shaped beads by dropping alginate solution into crosslinking agent solution, which is mainly studied as an adsorbent in aqueous systems and drug delivery carriers [48,49]. Even alginate encapsulation has many advantages, but it has not been used as an adsorbent encapsulation polymer with clay or PCH for atmospheric pollutant adsorption. 

In this study, PCH based on bentonite and alginate-PCH beads (Alg-PCH) was successfully prepared. From the powder X-ray diffraction (PXRD) patterns and transmission electron microscopy (TEM) images, the characteristic *c*-axis stacking order of the layers was disordered due to the organic moieties into the interlayer space of bentonite. The N_2_ adsorption–desorption analysis results show that SSA and the pore volume of PCH and Alg-PCH were enhanced compared to parent bentonite. Additionally, Alg-PCH shows a more enhanced thermal stability than alginate powder and alginate beads without PCH from thermal gravimetric analysis. The Alg-PCH preparation condition was optimized by control alginate concentration, alginate:PCH weight ratio, and concentration of CaCl_2_ solution, after which the toluene adsorption capacity was evaluated. Moreover, three different sizes of Alg-PCH bead were prepared by changing dropping tips, and the following toluene adsorption–desorption experiments show size-dependent toluene adsorption–desorption capacity according to bead size. Furthermore, the calculated toluene adsorption capacity of Alg-PCH was determined as 64.7 mg/g, which was around two times higher than the metal organic framework, which has similar SSA. 

## 2. Materials and Methods

### 2.1. Materials

Bentonite, hexadecyl trimethyl ammonium bromide (HDTMAB), and dodecylamine (DDA) were obtained from Sigma-Aldrich Co. LLC. (St. Louis, MO, USA). Tetraethyl orthosilicate (TEOS) was purchased from Daejung Chemicals & Metals Co. LTD. (Siheung-si, Gyeonggi-do, South Korea). Alginate was obtained from Junsei Chemical Co., Ltd. (Chuo-ku, Tokyo, Japan). Calcium chloride (CaCl_2_) was acquired from Dong Yang Chemical Co., Ltd. (Yeongnam-gun, Jeollanam-do, South Korea). Toluene (C_7_H_8_) was obtained from Duksan Pure Chemicals (Ansan-si, Gyeonggi-do, South Korea). All chemicals were used without further purification. 

### 2.2. Preparation of PCH

PCH based on bentonite was prepared by a modified method from our previous report [50]. For the practical application of Alg-PCH, experimental scales of Alg-PCH were set based on the 30 g of bentonite. First, bentonite and HDTMAB were dispersed into deionized water in the ratio of 1 g:0.4009 g:50 mL, and the amount of HDTMAB was calculated by 100% cation exchange capacity of bentonite (110 meq/100 g) [51]. The obtained suspension was stirred with a magnetic stirrer (300 rpm) at 60 °C for 24 h. The product (HDTMAB-Bt) was centrifuged (3000 rpm for 10 min) and washed several times with deionized water and then dried in the oven at 60 °C. The solid product of HDTMAB-Bt was ground with a mortar and then sieved until 150–500 μm particle size was obtained.

The PCH was synthesized by mixing the HDTMAB-Bt, DDA, and TEOS in the weight ratio of 1:1:60. The mixture was stirred with a magnetic stirrer (300 rpm) for 4 h at room temperature. The final product was collected by centrifugation (3000 rpm for 10 min) and washed several times with deionized water. Then sample was dried in the oven at 60 °C for 24 h and ground with a mortar, and 150–500 µm particles were collected for further calcination. Finally, powdered product was calcined at 550 °C for 6 h with temperature increasing rate of 3 °C/min. 

### 2.3. Encapsulation of PCH Using Alginate 

Alginate-PCH beads (Alg-PCH) were simply prepared by dropping alginate/PCH mixed slurry into CaCl_2_ solution. To optimize preparation condition of Alg-PCH, various concentrations of alginate, weight ratios (alginate:PCH), and concentrations of CaCl_2_ solution were utilized. The various alginate concentrations based on the weight ratio between PCH and alginate (500, 250, 150, 100, and 83.3 mg for 1:10, 1:20, 1:40, 1:50, and 1:60, respectively) were controlled. For this experiment, 5 g of PCH was dispersed into prepared alginate solution and stirred for 1 h with an overhead stirrer. After that, mixed solutions of PCH and alginate were transferred to 10 mL syringes with 3 mm tip size then dropped into CaCl_2_ solution by a syringe pump (NE4000, NEW ERA) at 1.5 mL/min under vigorous stirring. After 30 min, prepared Alg-PCHs were washed three times with deionized water and then dried in a drying oven at 40 °C for 12 h. Obtained dried Alg-PCHs were re-dried in a vacuum oven at 135 °C for 3 h for activation and dehydration of samples. 

According to the previous report, the alginate concentration increases, and the viscosity and surface tension also increase, which is not suitable for the formation of stable beads [52]. The three different alginate concentration (0.5%, 1.0%, and 1.5%; *w*/*v*) were utilized with the maximum PCH content to optimize the method for the preparation of Alg-PCH. To investigate the effect of the concentration of CaCl_2_ solution on bead formation, alginate concentration and weight ratio of alginate:PCH were fixed as 0.5% (*w*/*v*) and 1:50, respectively, based on results from earlier sections. To optimize Alg-PCH preparation conditions, three different CaCl_2_ concentrations (2.0, 3.0 and 4.0 *w*/*v*% for 0.18, 0.27 and 0.36 M, respectively) were utilized, and the procedure for Alg-PCH was the same as describe above. 

After optimization of the methodology for Alg-PCH formation, bead sizes were controlled by various sizes of extrusion tip (2, 3, and 4 mm) with optimized experimental conditions as above. Obtained dried bead size was measured by ImageJ software (National Institute of Health and the Laboratory for Optical and Computational Instrumentation (University of Wisconsin), Madison, WI, USA) to calculate pixel to mm.

### 2.4. Characterization of Alginate PCH Bead

According to the optimization experiments, the Alg-PCH for detailed characterization was selected by the following reaction condition: 0.5% (*w*/*v*) of alginate, 2% (0.18 M) of CaCl_2_ solution, a 1:50 alginate:PCH weight ratio, and a 3 mm extrusion tip, respectively. 

The PXRD patterns were collected in the range from 5° to 80°, utilizing Bruker DE/D8 Advance (Bruker AXS GmbH, Berlin, Germany) with a 5 mm air-scattering slit, a 2.6 mm equatorial slit, and time step increments of 3.9°/min. Fourier transformed infrared (FT-IR) spectra in the range of 650–4000 cm^−1^ were acquired with a Jasco FT/IR-4100 spectrometer (Jasco International Co. Ltd., Tokyo, Japan) with 32 scans and a 4 cm^−1^ resolution. The thermo-gravimetric analysis (TGA) and differential thermal analysis (DTA) were carried out with DTG-60 (Shimadzu, Kyoto, Japan) using and alumina crucible at a heating rate of 10 °C/min in the range of 25–800 °C. The N_2_ adsorption–desorption isotherm curves and Brunauer–Emmet–Teller (BET) surface area were obtained by a 3Flex physisorption analyzer (Micromeritics, Norcross, GA, USA). The average pore volume and size were determined using the Barrett–Joyner–Halenda (BJH) method. The microscopic images of bentonite, PCH, and Alg-PCH were collected by TEM (JEM-2100F, Jeol, Tokyo, Japan) utilizing a 200 kV accelerated electron beam. To prepare the TEM specimen, powdered bentonite, PCH, and grounded Alg-PCH were dispersed into deionized water (approximately 1 mg/mL) and then ultrasonicated for 15 min. A drop of suspension was placed on the 200 square mesh copper grid with carbon film then dried in the oven at 60 °C. To observe the morphology of Alg-PCH without grounding, the bead sample was subjected to block preparation with Spurr’s resin. The prepared blocks were sectioned using an ultramicrotome (EM UC7, Leica Microsystems, Wetzlar, Germany) and then placed on the 200 square mesh copper grid with carbon film.

### 2.5. Toluene Adsorption–Desorption Experiments

The toluene adsorption experiment was conducted by following our previous report [50], and a gas chromatography-flame ionization detector (GC-FID; YL6500, Youngin, South Korea) was utilized for quantification of toluene. For adsorption experiments, the designed concentration of toluene gas (1000 ppm) was produced by toluene solution. Liquid toluene was injected with a syringe pump into the airstream for vaporization then flowed into a chamber at 1500 mL/min air flow rate by a mass flow controller (space velocity 15,929 h^−1^). For adsorption experiments, toluene gas was used after 30 min stabilization in a chamber and then flowed to the column (3 cm ø × 26 cm) packed with 2 g of prepared sample at the designed flow rate. The toluene adsorption experiments were terminated when the toluene concentration had not changed for 10 mins (within 3% of changes) by GC-FID with the following conditions: oven temperature of 230 °C, flow rate of 3 mL/min, split ratio of 1:5, and FID temperature of 250 °C. Desorption experiments were conducted immediately after adsorption experiments following 30 minutes stabilization of GC-FID by air purging. After stabilization, the air was flowed at 1500 mL/min flow rate by a mass flow controller into a column packed with 2 g of adsorbed sample at room temperature for 40 min. The desorption efficacy of adsorbents was obtained with GC-FID at the same conditions of adsorption. The adsorption and desorption capacity was calculated from the amount of non-adsorbed toluene from GC-FID quantitative results. 

## 3. Results

### 3.1. Characterization of Bentonite, PCH, and Alg-PCH

#### 3.1.1. Physicochemical Property Analysis

**Figure 1:**
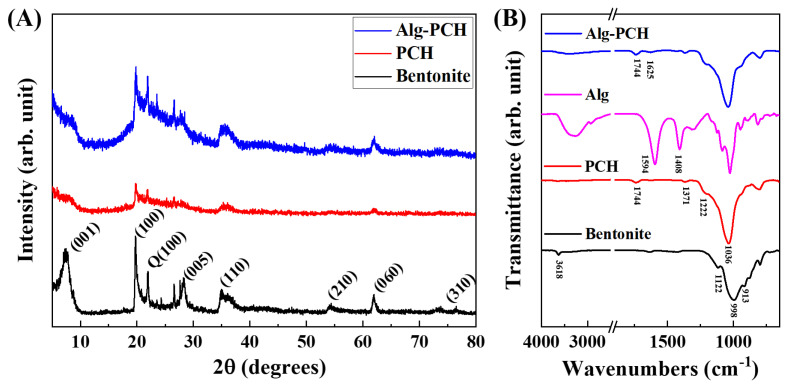
(**A**) Powder X-ray diffraction patterns of bentonite, PCH, and Alg-PCH (Q; quartz) and (**B**) FT-IR spectra of bentonite, PCH, alginate, and Alg-PCH.

The crystal structural changes of PCH and Alg-PCH based on bentonite were investigated by PXRD as shown in Figure 1A. Bentonite shows characteristic diffractions at 7.46°, 19.72°, 28.26°, 35.07°, 54.27°, 61.95°, and 76.44° for (001), (100), (005), (110), (210), (060), and (310), respectively, as previously reported [53]. In the case of PCH, the intensity of characteristic diffractions, such as (001), which corresponded to the *c*-axis stacking order of the layer structure of bentonite, was dramatically decreased (red line in Figure 1A). This decrement in diffractions might be attributed to the introduction of organic moieties (TEOS and DDA) into the interlayer space of bentonite. From diffraction patterns from powdered Alg-PCH (1:50 for alginate:PCH with 2% (0.18 M) of CaCl_2_), intensity of most diffractions including (001) and (110) was slightly increased compared to PCH (blue line in Figure 1). This phenomenon might be attributed to the polymerization of alginate with CaCl_2_, which improves the stacking order of bentonite layers [54,55]. From PXRD analysis, the crystal structure of PCH was not significantly changed after the introduction of organic moieties for porous heterostructure. 

The FT-IR spectra were collected to investigate the chemical property changes of bentonite, PCH, and Alg-PCH (Figure 1B). From the FT-IR spectra of parent bentonite, characteristic Si-O, Al-Al-OH, and Si-O-Mg bending vibrations were observed in the range of 998–1116, 913, and around 800 cm^−1^, respectively. Furthermore, Al-Al-OH stretching vibration was observed at 3618 cm^−1^, which was well matched with the previous report [53]. After introducing organic moieties (HDTMA and TEOS), the strong vibration attributed to Si-O at 998 cm^−1^ shifted to 1036 cm^−1^ due to the increment in amorphous silica contents during the preparation of PCH [56]. Additionally, the new vibrations were developed at 1222, 1371, and 1744 cm^−1^ which were assigned as C-N, aliphatic C-H, and C-O vibrations by introducing HDTMA, respectively [57]. The alginate powder shows characteristic asymmetric and symmetric stretching vibration of the carboxylate group (COO^−^) in alginate at 1408 and 1594 cm^−1^. After polymerization of alginate by CaCl_2_ with PCH, most vibrations coming from PCH were observed, and C=O vibrations were observed at 1625 cm^−1^, which developed by ionic bonding between calcium ions and alginate [58]. According to the PXRD and FT-IR spectra, the organic moieties (HDTMA and TEOS) were well incorporated into bentonite for the porous heterostructure (PCH), and also bead formation with alginate and CaCl_2_ (Alg-PCH) did not significantly change the crystal structure of PCH.

#### 3.1.2. Pore Structure and Morphological Analysis

**Figure 2:**
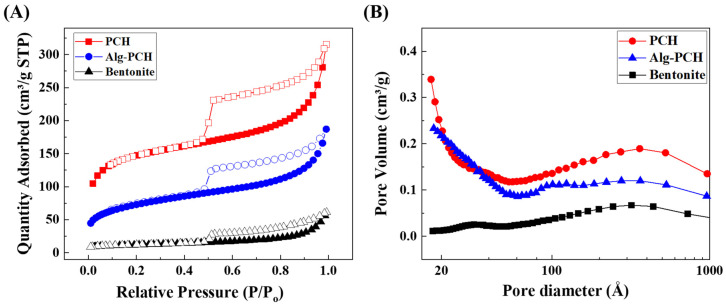
(**A**) N_2_ adsorption–desorption hysteresis loop and (**B**) pore volume distribution graph of bentonite, PCH, and Alg-PCH.

Nitrogen adsorption–desorption experiments were carried out to investigate the SSA and pore structure of parent bentonite, PCH, and Alg-PCH (Figure 2 and Table 1). From the N_2_ adsorption–desorption hysteresis loop shown in Figure 2A, all samples show type IV, which is attributed to capillary condensation that occurs in mesopores (2–50 nm) [59]. The obtained SSA of parent bentonite, PCH, and Alg-PCH was determined as 43, 538, and 256 m^2^/g, respectively. It is worth noting that SSA was increased around 12.5 times after the introduction of organic moieties, which was attributed to the silicon component of TEOS as a pillar-like structure. The SSA of Alg-PCH was decreased by ~50% after complexation with alginate, but still had a ~6 times higher value than parent bentonite and similar values to the alginate–metal organic framework (MOF) hybrid [59]. This decrement in SSA of Alg-PCH was attributed to the polymerization of alginate on the surface of PCH. Furthermore, the detailed pore size and pore volume calculated by the BJH method are summarized in Table 1. The pore volume and size of bentonite were determined as 0.087 cm^3^/g and 13.46 mm, respectively, which is quite well matched with a previous report on Na^+^-dominant bentonite [60]. Otherwise, PCH and Alg-PCH show around 3.8- and 2.8-fold higher pore volume, respectively, than parent bentonite (Figure 2B). The higher pore volume of PCH than parent bentonite was attributed to the porous structure organized with organic moieties (TEOS and DDA). 

The pore volume distribution graphs of all samples lie in the region from 10 to 100 nm. In the case of Alg-PCH, around 10 nm of pores observed might be due to the pore forming during the polymerization of alginate beads. Interestingly, the pore volume of Alg-PCH was decreased by around 27%, while SSA decreased by ~50% after complexation with alginate. In addition, the pore size of Alg-PCH was decreased by only 12%, which indicates the preservation of pore size even after the polymerization with alginate. From the N_2_ adsorption–desorption analysis, the introduction of porosity with TEOS and DDA into bentonite dramatically increased SSA and pore volume, and polymerization with alginate to form Alg-PCH did not affect to pore volume and pore size significantly.

**Figure 3:**
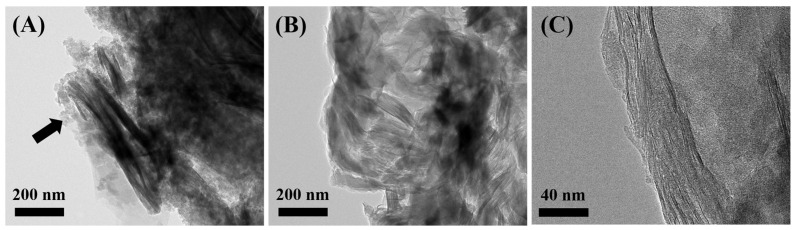
Transmittance electron microscopy images of (**A**) powdered Alg-PCH; (**B**,**C**) sectioned Alg-PCH fixed with Spurr’s resin.

To investigate the morphological changes of parent bentonite, PCH, and Alg-PCH, TEM analysis was carried out (Figure 3 and Figure S1). As shown in Figure S1, parent bentonite shows a characteristic well-ordered layered structure [50,61]. After introducing organic moieties into the bentonite layer, the well-ordered layered structure was transformed into a disordered structure, which corresponds well with PXRD results (Figure 1). After the polymerization of PCH with alginate, bentonite layers were re-ordered compared to PCH and alginate polymer (black arrow in Figure 3A is the covered PCH). To evaluate the detailed layered structure in Alg-PCH, a TEM specimen was prepared with Spur’s resin and sectioned by ultramicrotome (Figure 3B, C). From the TEM images of the sectioned Alg-PCH, the characteristic PCH layers show a better organized structure than PCH itself, which is also well matched with the increment in diffraction patterns of (001) attributed to the *c*-axis stacking of bentonite.

#### 3.1.3. Thermal Analysis

**Figure 4:**
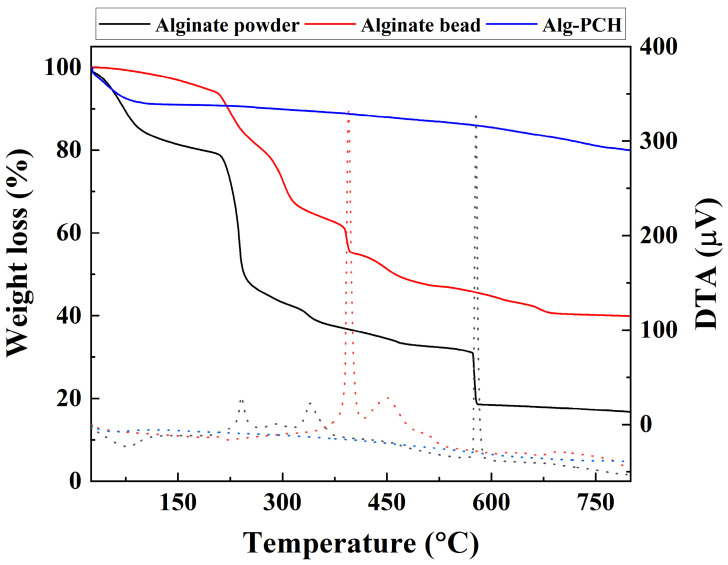
Thermal gravimetric analysis (TGA; solid line) and differential thermal analysis (DTA; dashed line) results of alginate powder, powdered alginate bead, and Alg-PCH.

The thermal stability of alginate is also an important factor for its use as an adsorbent for VOCs in laundry facilities due to the high temperature gases produced. Thermal gravimetric and differential thermal analyses were carried out with alginate powder, powered alginate beads, and Alg-PCH (Figure 4). The alginate powder showed three steps of thermal decomposition in the range of 25–100 °C, 100–250 °C, and above 250 °C. From DTA results, the first endothermic peak appearing at around 75 °C was probably attributed to the water loss of hydrophilic functional polymeric groups and the following exothermic peaks appeared around 240, 339, and 577 °C, corresponding to the polymer degradation and decomposition [62]. After polymerization with CaCl_2_, the characteristic exothermic peaks of alginate were shifted to higher temperatures from 240 and 339 °C to 394 and 450 °C, respectively. In addition, the weight loss difference of alginate and alginate beads was approximately 24%, which also supported the enhancement of thermal stability of alginate. The TG and DTA curve of Alg-PCH showed a 10% weight loss without characteristic exothermic peaks of alginate. Considering the weight loss of surface water of the sample (around 10% at 100 °C) and PCH itself, thermal stability of Alg-PCH showed enhanced thermal stability. 

### 3.2. Optimization of Alg-PCH Preparation Condition for Toluene Adsorption

#### 3.2.1. Alginate Concentration and Alg:PCH Ratio

**Figure 5:**
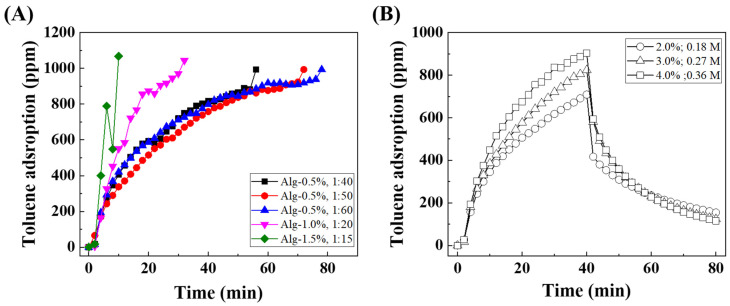
(**A**) Toluene adsorption curve depending on alginate concentration and weight ratio between alginate and PCH; (**B**) toluene adsorption–desorption curve of Alg-PCH depending on concentration of CaCl_2_ solution (circle; 2%, triangle; 3% and square; 4%) with 40 min cycle.

To optimize the toluene adsorption properties of Alg-PCH, first, the concentration of alginate and amount of PCH were controlled because the alginate concentration and alginate: adsorbent weight ratio could affect the SSA of alginate beads (Table 2) [63]. The toluene adsorption capacities on Alg-PCH were determined by fixing the alginate concentration (0.5%) with CaCl_2_ (2%) solution, and controlled amounts of PCH, 10, 20, 40, 50, and 60 times the amount of alginate (fixed as 1), are displayed in Figure 5A. In the case of low amount of PCH (1:10 and 20), Alg-PCH was not formed or formed irregular beads, while higher PCH amount (1:40, 50, and 60) developed a well-formed sphere shape of Alg-PCH. According to this result, the amount of PCH was affected by the formation of alginate beads, and at least 1:40 of alginate and PCH ratio is required for the formation of Alg-PCH. 

From these results, two different alginate concentrations were utilized for further optimization. In the case of a concentration of alginate solution higher than 0.5%, a high weight ratio between alginate and PCH (1:30 for 1.0% and 1:20% for 1.5%) did not form a sphere shape of Alg-PCH. Higher alginate concentration and PCH content ratio make the viscosity of the solution stronger, which increases the surface tension of the mixture and does not form regular water droplet beads [52]. Interestingly, when the concentration of alginate was increased, the amount of required PCH for Alg-PCH was decreased. From these optimization results, the formation of Alg-PCH was highly dependent on the alginate concentration and amount of PCH. 

To determine the toluene adsorption capacity, toluene column adsorption experiments were carried out with prepared Alg-PCH along the concentration of alginate and amount of PCH (Figure 5A). The calculated toluene adsorption capacity of each sample is displayed in Table 2. The toluene adsorption capacity of Alg-PCH gradually decreased with the increment in alginate concentration, which might be due to blocking of the SSA and pores in PCH. It is noteworthy that the amount of PCH did not correspond to the toluene adsorption capacity, and the optimal conditions for preparation of Alg-PCH were determined to be 0.5% of alginate and a 1:50 ratio of alginate and PCH, which shows 71.86 mg/g.

#### 3.2.2. CaCl_2_ Concentration

The concentration of calcium chloride (CaCl_2_) solution is also an important factor for the preparation of alginate beads. To optimize the preparation condition for Alg-PCH, three different CaCl_2_ concentrations (2%, 3%, and 4% refer to 0.18, 0.27, and 0.36 M, respectively) with optimized reaction conditions from the previous section (0.5% alginate, a 1:50 alginate:PCH) were utilized. In this experiment, the toluene adsorption–desorption experiments each took 40 min, according to the consuming time for dry cleaning. As shown in Figure 5B, calculated adsorption capacities were determined as 61.13, 53.34, and 44.55 mg/g for 2%, 3%, and 4%, respectively (Table 3). On the other hand, the desorption performance showed the opposite trend compared to adsorption capacities. In the case of 4% CaCl_2_, it adsorbed the lowest amount of toluene, but the desorption performance (~71.5%) was the highest. From this result, a high concentration of CaCl_2_ leads to a high polymerization degree of alginate on the surface of PCH, which might be decreased by pore size or blocked pores for toluene adsorption. The toluene adsorption and desorption performance might be affected by the concentration of CaCl_2_, which enhanced polymerization due to Ca^2+^ acting as a cross-linking agent. The enhanced polymerization led to the collapse of the micropores in the PCH structure; therefore, low adsorption capacity was expected. Moreover, the better desorption performance was also expected since the desorption from the micropore has been reported to be more difficult than that from the mesopore or macropore [28,64]. However, it should be noted that the desorption was performed by just purging air without vacuum or heating; therefore, higher desorption and recovery could be expected when applying vacuum or heating in further study [28,65].

From these results, the optimal condition for Alg-PCH was chosen as 0.5% alginate, a 1:50 alginate:PCH (*w*/*w*), and 2% (0.18 M) of CaCl_2_ solution for further experiments.

#### 3.2.3. Bead Size

**Figure 6:**
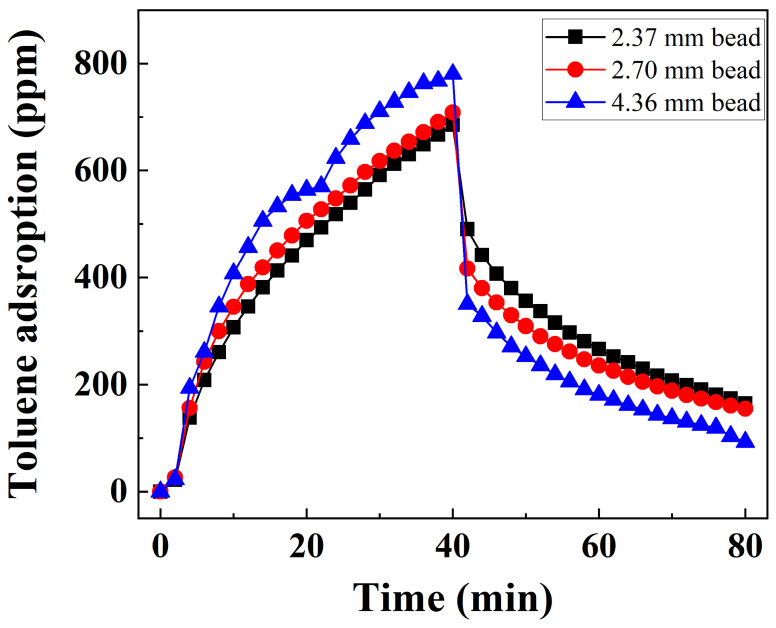
Toluene adsorption–desorption curve of Alg-PCH depending on the bead size with 40 min cycle.

To determine the bead size effect of Alg-PCH for toluene adsorption–desorption experiments, three different extrusion tips (2, 3, and 4 mm) were utilized (Figure 6). The average diameter of Alg-PCH obtained from three different tip sizes was determined as 2.37 ± 0.13, 2.70 ± 0.09, and 4.36 ± 0.32 mm, respectively, by photographs of beads and calculated by Image J software (Figure S2). As shown in Figure 6, calculated adsorption capacities were determined as 64.65, 61.13, and 53.66 mg/g for increasing bead sizes, respectively. The highest adsorption capacity of prepared Alg-PCH with optimized conditions (64.65 mg/g) shows around 74% of granular activated carbon, approximately two times higher than MOF powder, with similar SSA and granular silica gel, respectively (Table 4) [66,67,68]. On the other hand, desorption capacities were determined as 35.37, 29.66, and 25.44 mg/g, respectively. The percentages of the desorbed amount compared to adsorbed amount were also decreased, 54.7%, 48.5%, and 47.4% for increasing bead sizes. The higher toluene adsorption–desorption capacity of small Alg-PCH beads than bigger ones might be attributed to the large SSA of beads when they are packed in the column. Additionally, smaller bead could contribute to the high flow resistance of the column because of pressure drop. A 2.37 mm bead can adsorb toluene in the condition of 1500 mL/min flow rate, which is calculated to 15,929 h^−1^ of space velocity in the column, showing higher space velocity than other adsorption literature [69,70].

## 4. Conclusions

PCH based on the bentonite was successfully prepared utilizing organic moieties, TEOS and DDA. From the structural and N_2_ adsorption–desorption analysis, PCH shows disordered *c*-axis layered stacking and 12.5-fold enhanced SSA, with a 3.8-fold higher pore volume. After the formation of Alg-PCH, the pore volume and specific surface area of PCH were decreased due to alginate polymerization as a result of re-stacking layers. Even for the decrement in specific surface area and pore volume, Alg-PCH showed approximately 6- and 2.8-fold increases compared to parent bentonite. The thermal stability of Alg-PCH was examined with thermal gravimetric analysis, and PCH which crosslinked with alginate showed higher thermal stability than alginate itself. From the TEM images, the characteristic bentonite layers were disordered after the introduction of porosity and then re-stacked following polymerization with alginate. The concentration of alginate, CaCl_2_ solution, and weight ratio between PCH and alginate was controlled to optimize reaction conditions. According to the toluene adsorption–desorption experiments, the reaction conditions for Alg-PCH were selected as 0.5% of alginate and 2% of CaCl_2_ solution with a 1:50 alginate:PCH weight ratio. The bead size of optimized Alg-PCH also showing an effect on toluene adsorption–desorption might be due to the surface area difference of Alg-PCH when packed in the column. The toluene adsorption and desorption capacities of optimized Alg-PCH were determined to be 64.65 and 35.57 mg/g, receptively. The adsorption capacity on optimized Alg-PCH showed around 74% of granular activated carbon, two times higher than MOF, which has similar SSA and granular silica gel. From this research, Alg-PCH could be utilized as promising VOC adsorbents not only in laundry facilities but also in industrial fields under harsh conditions. 

## Figures and Tables

**Table 1:** Brunauer–Emmet–Teller (BET) surface area and pore analysis results for bentonite, PCH, and Alg-PCH.

Sample	BET Surface Area (m^2^/g)	BJH Pore Volume (cm^3^/g)	BJH Pore Size (nm)
Bentonite	43	0.087	13.46
PCH	538	0.336	7.32
Alg-PCH	256	0.242	6.44

**Table 2:** Detailed information of optimization of Alg-PCH with toluene adsorption capacity.

Alginate Concentration (%)	Alginate:PCH (*w*/*w*)	Adsorption Capacity (mg/g)	Formation
0.5%	1:10	N.D.	×
	1:20	N.D.	△
	1:40	59.13	○
	1:50	71.86	○
	1:60	64.73	○
1.0%	1:20	31.43	○
	1:30	N.D.	×
1.5%	1:15	15.32	○
	1:20	N.D.	×

**Table 3:** Summarized toluene adsorption–desorption capacity depending on CaCl_2_ concentration.

CaCl_2_ Concentration (*w*/*v*)	Adsorption Capacity (mg/g)	Desorption Capacity (mg/g)	Desorption Efficacy (%)
2%	61.63	29.66	48.5
3%	53.34	27.64	51.9
4%	44.55	31.87	71.5

**Table 4:** Summarized toluene adsorption capacities and BET surface area of various adsorbents.

Sample	Fixed Bed Adsorption		BET Surface Area (m^2^/g)	Ref.
	Q_ad_ (mg/g)	P_r_ (P/P_0_)		
Granular activated carbon	87.9	0.045	-	[66]
Granular silica gel	37.5	0.045	-	[66]
Granular 13X zeolite	7.9	0.091	-	[66]
Al-Mt@C powder	39.9	0.029	163	[67]
MOF-5 powder	32.9	0.0026	424	[68]
MIL-101(Fe) powder	98.3	0.0026	377	[68]
PCH powder	199.7	0.029	538	This study
Alg-PCH bead	64.7	0.029	256	This study

## Data Availability

The data presented in this study are available on request from the corresponding author.

## Supplementary Materials

The following are available online at https://www.mdpi.com/2079-4991/11/2/388/s1, Figure S1: Transmission electron microscopy images of bentonite and PCH; Figure S2: Converted photographs of Alg-PCH prepared by three different extrusion tips (A) 2, (B) 3, and (C) 4 mm with ImageJ software.

## References

[1] Wang H., Nie L., Li J., Wang Y., Wang G., Wang J., Hao Z. (2013). Characterization and assessment of volatile organic compounds (VOCs) emissions from typical industries. Chin. Sci. Bull..

[2] Fiore A.M., Naik V., Spracklen D.V., Steiner A., Unger N., Prather M., Bergmann D., Cameron-Smith P., Cionni I., Collins W.J. (2012). Global air quality and climate. Chem. Soc. Rev..

[3] Derwent R.G. (1995). Sources, distributions, and fates of VOCs in the atmosphere. Issues Environ. Sci. Technol..

[4] Bari M.A., Kindzierski W.B. (2018). Ambient volatile organic compounds (VOCs) in Calgary, Alberta: Sources and screening health risk assessment. Sci. Total Environ..

[5] An T., Huang Y., Li G., He Z., Chen J., Zhang C. (2014). Pollution profiles and health risk assessment of VOCs emitted during e-waste dismantling processes associated with different dismantling methods. Environ. Int..

[6] Jia C., Batterman S., Godwin C. (2008). VOCs in industrial, urban and suburban neighborhoods, Part 1: Indoor and outdoor concentrations, variation, and risk drivers. Atmos. Environ..

[7] United States Environmental Protection Agency Air Pollutant Emissions Trends Data. https://www.epa.gov/air-emissions-inventories/air-pollutant-emissions-trends-data.

[8] Li M., Liu H., Geng G., Hong C., Liu F., Song Y., Tong D., Zheng B., Cui H., Man H. (2017). Anthropogenic emission inventories in China: A review. Nat. Sci. Rev..

[9] Im J., Kim H., Kim M., Lee J., Lee S., Lee C. (2018). A Study on the Variation of Hazardous Pollutant Emissions in Korea from 2006 to 2015. J. Environ. Health Sci..

[10] Parrish D.D. (2006). Critical evaluation of US on-road vehicle emission inventories. Atmos. Environ..

[11] Unwin J., Cocker J., Scobbie E., Chambers H. (2006). An Assessment of Occupational Exposure to Polycyclic Aromatic Hydrocarbons in the UK. Ann. Occup. Hyg..

[12] Singh P., Chauhan S.R. (2016). Carbonyl and aromatic hydrocarbon emissions from diesel engine exhaust using different feedstock: A review. Renew. Sustain. Energy Rev..

[13] Samet J. (1990). Environmental Controls and Lung Disease: Report of the ATS Workshop on Environmental Controls and Lung Disease, Santa Fe, New Mexico, March 24–26, 1988. Am. Rev. Respir..

[14] Yuan B., Shao M., Lu S., Wang B. (2010). Source profiles of volatile organic compounds associated with solvent use in Beijing, China. Atmos. Environ..

[15] Li X., Zhang L., Yang Z., Wang P., Yan Y., Ran J. (2020). Adsorption materials for volatile organic compounds (VOCs) and the key factors for VOCs adsorption process: A review. Sep. Purif. Technol..

[16] Chuang C.L., Chiang P.C., Chang E.E. (2003). Modeling VOCs adsorption onto activated carbon. Chemos. Oxford.

[17] Detchanamurthy S., Gostomski P.A. (2012). Biofiltration for treating VOCs: An overview. Rev. Environ. Sci. Bio. Technol..

[18] Parthasarathy G., El-Halwagi M. (2000). Optimum mass integration strategies for condensation and allocation of multicomponent VOCs. Chem. Eng. Sci..

[19] Tichenor B.A., Palazzolo M.A. (1987). Destruction of volatile organic compounds via catalytic incineration. Environ. Prog..

[20] Ruhl M.J. (1993). Recover VOCs via adsorption on activated carbon. Chem. Eng. Prog..

[21] Gupta V.K., Verma N. (2002). Removal of volatile organic compounds by cryogenic condensation followed by adsorption. Chem. Eng. Sci..

[22] Khan F.I., Ghoshal A.K. (2000). Removal of volatile organic compounds from polluted air. J. Loss Prev. Process Ind..

[23] Zhang X., Gao B., Creamer A.E., Cao C., Li Y. (2017). Adsorption of VOCs onto engineered carbon materials: A review. J. Hazard. Mat..

[24] Djilani C., Zaghdoudi R., Modarressi A., Rogalski M., Djazi F., Lallam A. (2012). Elimination of organic micropollutants by adsorption on activated carbon prepared from agricultural waste. Chem. Eng. J..

[25] Nasrullah A., Bhat A.H., Naeem A., Isa M.H., Danish M. (2018). High surface area mesoporous activated carbon-alginate beads for efficient removal of methylene blue. Int. J. Biol. Macromol..

[26] Cazetta A.L., Junior O.P., Vargas A.M.M., Da Silva A.P., Zou X., Asefa T., Almeida V.C. (2013). Thermal regeneration study of high surface area activated carbon obtained from coconut shell: Characterization and application of response surface methodology. J. Anal. App. Pyrolysis.

[27] Jarraya I., Fourmentin S., Benzina M., Bouaziz S. (2010). VOC adsorption on raw and modified clay materials. Chem. Geol..

[28] Yang X., Yi H., Tang X., Zhao S., Yang Z., Ma Y., Feng T., Cui X. (2018). Behaviors and kinetics of toluene adsorption-desorption on activated carbons with varying pore structure. J. Environ. Sci..

[29] Takahashi N., Ushiki I., Hamabe Y., Ota M., Sato Y., Inomata H. (2016). Measurement and prediction of desorption behavior of five volatile organic compounds (acetone, n-hexane, methanol, toluene, and n-decane) from activated carbon for supercritical carbon dioxide regeneration. J. Supercrit. Fluids.

[30] Zaitan H., Bianchi D., Achak O., Chafik T. (2008). A comparative study of the adsorption and desorption of o-xylene onto bentonite clay and alumina. J. Hazard. Mat..

[31] Qu F., Zhu L., Yang K. (2009). Adsorption behaviors of volatile organic compounds. J. Hazard. Mat..

[32] Pires J., Bestilleiro M., Pinto M., Gil A. (2008). Selective adsorption of carbon dioxide, methane and ethane by porous clays heterostructures. Sep. Purif. Technol..

[33] Bellir K., Lehocine M.B., Meniai A.-H. (2013). Zinc removal from aqueous solutions by adsorption onto bentonite. Desalin. Water Treat..

[34] Okada T., Seki Y., Ogawa M. (2014). Designed nanostructures of clay for controlled adsorption of organic compounds. J. Nanosci. Nanotechnol..

[35] Celis R., Hermosin M.C., Cornejo J. (2000). Heavy metal adsorption by functionalized clays. Environ. Sci. Technol..

[36] Gier S., Johns W.D. (2000). Heavy metal-adsorption on micas and clay minerals studied by X-ray photoelectron spectroscopy. App. Clay Sci..

[37] Chen H., Koopal L.K., Xiong J., Avena M., Tan W. (2017). Mechanisms of soil humic acid adsorption onto montmorillonite and kaolinite. J. Colloid Interface Sci..

[38] Arellano-Cárdenas S., Gallardo-Velázquez T., Osorio-Revilla G., López-Cortéz M.D.S., Gómez-Perea B. (2005). Adsorption of Phenol and Dichlorophenols from Aqueous Solutions by Porous Clay Heterostructure (PCH). J. Mex. Chem. Soc..

[39] Cecilia J.A., García-Sancho C., Franco F. (2013). Montmorillonite based porous clay heterostructures: Influence of Zr in the structure and acidic properties. Microporous Mesoporous Mat..

[40] Rezaei F., Webley P. (2009). Optimum structured adsorbents for gas separation processes. Chem. Eng. Sci..

[41] Shim W.G., Lee J.W., Moon H. (2006). Adsorption equilibrium and column dynamics of VOCs on MCM-48 depending on pelletizing pressure. Microporous Mesoporous Mat..

[42] Küsgens P., Zgaverdea A., Fritz H.G., Siegle S., Kaskel S. (2010). Metal-organic frameworks in monolithic structures. J. Am. Ceram. Soc..

[43] Zaitseva N., Zaitsev V., Walcarius A. (2013). Chromium(VI) removal via reduction–sorption on bi-functional silica adsorbents. J. Hazard. Mat..

[44] Suchithra P.S., Vazhayal L., Peer Mohamed A., Ananthakumar S. (2012). Mesoporous organic–inorganic hybrid aerogels through ultrasonic assisted sol–Gel intercalation of silica–PEG in bentonite for effective removal of dyes, volatile organic pollutants and petroleum products from aqueous solution. Chem. Eng. J..

[45] Samiey B., Cheng C.-H., Wu J. (2014). Organic–Inorganic hybrid polymers as adsorbents for removal of heavy metal ions from solutions: A review. Materials.

[46] Zhao G., Huang X., Tang Z., Huang Q., Niu F., Wang X. (2018). Polymer-based nanocomposites for heavy metal ions removal from aqueous solution: A review. Polymer Chem..

[47] Lee K.Y., Mooney D.J. (2012). Alginate: Properties and biomedical applications. Prog Polymer Sci..

[48] Kwon O.-H., Kim J.-O., Cho D.-W., Kumar R., Baek S.H., Kurade M.B., Jeon B.-H. (2016). Adsorption of As(III), As(V) and Cu(II) on zirconium oxide immobilized alginate beads in aqueous phase. Chemosphere.

[49] Tønnesen H.H., Karlsen J. (2002). Alginate in drug delivery systems. Drug Dev. Ind. Pharm..

[50] Yang P., Song M., Kim D., Jung S.P., Hwang Y. (2019). Synthesis conditions of porous clay heterostructure (PCH) optimized for volatile organic compounds (VOC) adsorption. Korean J. Chem. Eng..

[51] Park J.-A., Kang J.-K., Kim J.-H., Kim S.-B., Yu S., Kim T.-H. (2015). Bacteriophage removal in various clay minerals and clay-amended soils. Environ. Eng. Res..

[52] Lee B.-B., Ravindra P., Chan E.-S. (2013). Size and Shape of Calcium Alginate Beads Produced by Extrusion Dripping. Chem. Eng. Technol..

[53] Alabarse F.G., Conceição R.V., Balzaretti N.M., Schenato F., Xavier A.M. (2011). In-situ FTIR analyses of bentonite under high-pressure. App. Clay Sci..

[54] Mandal S., Patil V.S., Mayadevi S. (2012). Alginate and hydrotalcite-like anionic clay composite systems: Synthesis, characterization and application studies. Microporous Mesoporous Mat..

[55] He Y., Wu Z., Tu L., Han Y., Zhang G., Li C. (2015). Encapsulation and characterization of slow-release microbial fertilizer from the composites of bentonite and alginate. App. Clay Sci..

[56] Basir N.M., Lintang H.O., Endud S. (2015). Phosphotungstic acid supported on acid-leached porous kaolin for friedel-crafts acylation of anisole. J. Teknol..

[57] Gârea S.A., Mihai A.I., Vasile E., Nistor C., Sârbu A., Mitran R. (2016). Synthesis of new porous clay heterostructures: The influence of co-surfactant type. Mat. Chem. Phys..

[58] Kusuktham B., Prasertgul J., Srinun P. (2014). Morphology and Property of Calcium Silicate Encapsulated with Alginate Beads. Silicon.

[59] Ambroz F., Macdonald T.J., Martis V., Parkin I.P. (2018). Evaluation of the BET Theory for the Characterization of Meso and Microporous MOFs. Small Methods.

[60] Kaufhold S., Dohrmann R., Klinkenberg M., Siegesmund S., Ufer K. (2010). N2-BET specific surface area of bentonites. J. Colloid Interface Sci..

[61] Huff W.D., Whiteman J.A., Curtis C.D. (1988). Investigation of a K-Bentonite by X-Ray Powder Diffraction and Analytical Transmission Electron Microscopy. Clays Clay Miner..

[62] Pereira R., Tojeira A., Vaz D.C., Mendes A., Bártolo P. (2011). Preparation and characterization of films based on alginate and aloe vera. Int. J. Polymer Anal. Charact..

[63] Lee D.W. (2017). Formulation and Characterization of the Metal-Organic Compound UiO-66. Master’s Thesis.

[64] Zhang X., Yang Y., Lv X., Wang Y., Liu N., Chen D., Cui L. (2019). Adsorption/desorption kinetics and breakthrough of gaseous toluene for modified microporous-mesoporous UiO-66 metal organic framework. J. Hazard. Mat..

[65] Zhou C., Zhou K., Li H., Xu X., Liu B., Li H., Zeng Z., Ma W., Li L. (2020). Pressure swing adsorption properties of activated carbon for methanol, acetone and toluene. Chem. Eng. J..

[66] Wang C.-M., Chang K.-S., Chung T.-W., Wu H. (2004). Adsorption Equilibria of Aromatic Compounds on Activated Carbon, Silica Gel, and 13X Zeolite. J. Chem. Eng. Data.

[67] Liu C., Cai W., Liu L. (2018). Hydrothermal carbonization synthesis of Al-pillared montmorillonite@carbon composites as high performing toluene adsorbents. App. Clay Sci..

[68] Vellingiri K., Kumar P., Deep A., Kim K.-H. (2017). Metal-organic frameworks for the adsorption of gaseous toluene under ambient temperature and pressure. Chem. Eng. J..

[69] Stuckert N.R., Yang R.T. (2011). CO_2_ Capture from the Atmosphere and Simultaneous Concentration Using Zeolites and Amine-Grafted SBA-15. Environ. Sci. Technol..

[70] Lu X., He J., Xie J., Zhou Y., Liu S., Zhu Q., Lu H. (2020). Preparation of hydrophobic hierarchical pore carbon–Silica composite and its adsorption performance toward volatile organic compounds. J. Environ. Sci..

