# Supplementary material for: Porous Clay Heterostructure with Alginate Encapsulation for Toluene Removal
Source: Nanomaterials (Basel). 2021 Feb 3;11(2):388. doi: 10.3390/nano11020388 (PMC7913573; doi:10.3390/nano11020388)
Supplement: Supplementary file 1 [file nanomaterials-11-00388-s001.pdf]

Supporting information

A Study of optimization using porous clay heterostructure with alginate encapsulation for toluene removal

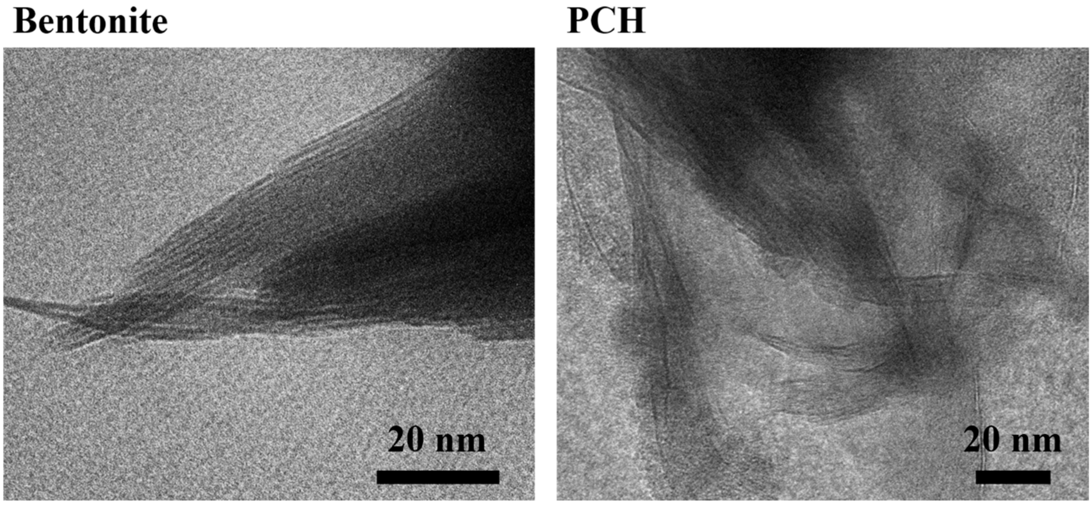

Figure S1. Transmission electron microscopy images of bentonite and PCH.

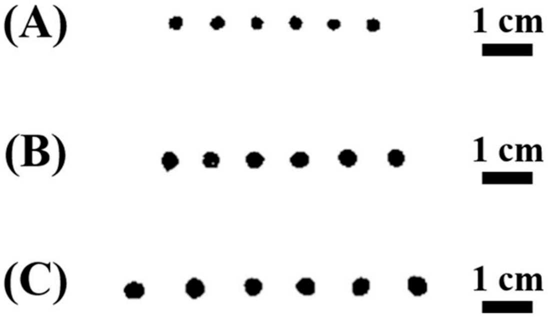

Figure S2. Converted photographs of Alg-PCH prepared by three different extrusion tips (A) 2, (B) 3 and (C) 4 mm with ImageJ software.
